# The Impact of Funding on Childhood TB Case Detection in Pakistan

**DOI:** 10.3390/tropicalmed4040146

**Published:** 2019-12-15

**Authors:** Amyn A. Malik, Hamidah Hussain, Jacob Creswell, Sara Siddiqui, Junaid F. Ahmed, Falak Madhani, Ali Habib, Aamir J. Khan, Farhana Amanullah

**Affiliations:** 1 Global Health Directorate, Indus Health Network, Karachi 75190, Pakistan; sara.siddiqui@ghd.ihn.org.pk (S.S.); junaid.fuad@ghd.ihn.org.pk (J.F.A.); falak.madhani@ghd.ihn.org.pk (F.M.); 2 Interactive Research and Development (IRD) Global, Singapore 189677, Singapore; hamidah.hussain@ird.global (H.H.); aamir.khan@ird.global (A.J.K.); 3 Emory University Rollins School of Public Health, Atlanta, GA 30329, USA; 4 Stop TB Partnership, 1218 Le Grand-Saconnex, Switzerland; jacobc@stoptb.org; 5 Interactive Health Solutions, Karachi 75350, Pakistan; ali.habib@ihsinformatics.com; 6 The Indus Hospital, Karachi 75190, Pakistan; farhana.maqbool@ird.global

**Keywords:** pediatric TB, verbal screening, contact tracing, resources

## Abstract

This study is a review of routine programmatically collected data to describe the 5-year trend in childhood case notification in Jamshoro district, Pakistan from January 2013 to June 2018 and review of financial data for the two active case finding projects implemented during this period. The average case notification in the district was 86 per quarter before the start of active case finding project in October 2014. The average case notification rose to 322 per quarter during the implementation period (October 2014 to March 2016) and plateaued at 245 per quarter during the post-implementation period (April 2016 to June 2018). In a specialized chest center located in the district, where active case finding was re-introduced during the post implementation period (October 2016), the average case notification was 218 per quarter in the implementation period and 172 per quarter in the post implementation period. In the rest of the district, the average case notification was 160 per quarter in the implementation period and 78 during the post implementation period. The cost per additional child with TB found ranged from USD 28 to USD 42 during the interventions. A continuous stream of resources is necessary to sustain high notifications of childhood TB.

## 1. Introduction

Childhood TB diagnosis can be difficult and hence many children who develop TB are missed. Of the 10 million people who develop TB each year, 10% or 1 million are children. While national TB programs (NTP) do not report 34% of all incident cases, more than half of children with TB are believed to be missed, resulting in 233,000 deaths each year [1,2]. Modeling studies suggest that 96% of these deaths occur in children who do not access TB treatment [3].

Children with TB are often not diagnosed and reported because of limited capacity of frontline health providers [4,5], lack of dedicated child health services with experienced and appropriately trained clinicians [4], non-specific symptoms overlapping with other common childhood diseases [6], complex diagnostic algorithms [6,7], lack of a sensitive point of care test and technical resources [8], and minimal contact tracing activities [2].

In Pakistan, the estimated TB incidence in 2018 was 265 cases per 100,000 population with approximately 62,000 cases in children. Of the estimated childhood cases, about one in four cases were not notified to the national program [1].

Successful interventions to improve case detection among children have included systematic screening at outpatient departments of hospitals and general practitioners with studies from Pakistan showing that this can increase the case notification among children between 2.5 and 7 times [9,10].

In many high TB burden countries, the response to the epidemic is highly donor dependent. Periodic funding of targeted interventions can lead to increases in diagnosis and notification [11] with a hope that the increase will be sustained given the strengthened health system and capacity building. Recently, the United Nations held a High-Level Meeting on Ending TB (UNHLM), where heads of states committed to mobilize at least 13 billion dollars annually by 2022 for the sufficient and sustainable financing of the global TB response, and to diagnose and treat 3.5 million children with TB between 2018 and 2022 [12].

Our objective is to describe the 5 years trend in childhood case notification in a rural district in Sindh province of Pakistan before, during and after focused active case finding and contact tracing efforts with injection of resources. We sought to understand the impact and cost of finding a child with TB during periodic funding from external sources.

## 2. Materials and Methods

### 2.1. Setting and Study Design

This study is a review of programmatically collected case notification data to describe the 5 year trend in childhood case notification in Jamshoro district, Pakistan from January 2013 to June 2018.

As part of the Stop TB Partnership’s TB REACH wave 4 funding, an active case finding and contact tracing project in a district in rural Sindh was conducted between October 2014 and March 2016. The detailed methodology of this project and results are reported elsewhere [10]. Briefly, the intervention systematically screened all children in outpatient departments of four large public sector hospitals in Jamshoro district for symptoms of TB and conducted household contact tracing of adults and children diagnosed with TB at these facilities. Three of these four hospitals had pediatric TB specialists as part of their medical staff and were already reporting pediatric TB cases. No other center reported pediatric TB cases regularly. One of the four hospitals is a specialized chest treatment center, which treats both drug-susceptible and drug-resistant TB. Community health workers were recruited from the catchment area and trained to administer questionnaires to assess TB symptoms using a custom-built mHealth data collection application with decision support. All individuals with a high likelihood of TB disease were referred to a TB medical officer for free evaluation and testing. Adults with TB and guardians of children diagnosed with TB were also asked to bring their family members to the health facility for TB screening.

At the specialized chest center, active case finding was re-started through a Global Fund initiative in October 2016 by adding one doctor and one nurse and providing support for data collection. The nurse was trained to administer questionnaires to assess TB symptoms in children in outpatient department using a custom-built mHealth data collection application with decision support. All children with a high likelihood of TB were referred to the medical officer for further evaluation and free testing.

There were no other notable changes in the district during the five-year period being analyzed.

### 2.2. Data Collection

Age-disaggregated TB case notification data were extracted from the registers of the provincial TB program (PTP) from quarter 1, 2013 to quarter 2, 2018.

Financial data from the TB REACH project was extracted from the accounting system maintained by the finance department. We calculated the operational cost per child verbally screened and cost per TB patient diagnosed through active case finding at the specialized chest center during the implementation period. It included human resources, design, deployment and maintenance of electronic data collection systems and the laboratory tests. We employed two community health workers and one field supervisor exclusively for the intervention and a government employed doctor was incentivized to screen and treat additional children found through the project. The project bore the costs of chest X-rays, Xpert MTB/RIF, Acid Fast Bacilli (AFB) smear and other laboratory and radiological tests as required. Costs were incurred in Pakistani Rupees (PKR) and were converted to US dollars (USD) using the average 2015 exchange rate of 1 USD to 103.1 PKR.

Financial data from the Global Fund project for the support provided to the specialized chest center was extracted from the accounting system from October 2016 to June 2018. During this period, the facility employed one doctor and one nurse. A dedicated doctor was only employed for half of the time period. Chest x-rays, Xpert MTB/RIF, AFB smear and other laboratory and radiological tests as required were done free of charge for the patients. Costs were incurred in Pakistani Rupees (PKR) and were converted to US dollars (USD) using the average 2017 exchange rate of 1 USD to 105.3 PKR.

### 2.3. Analysis

**Figure 1:**
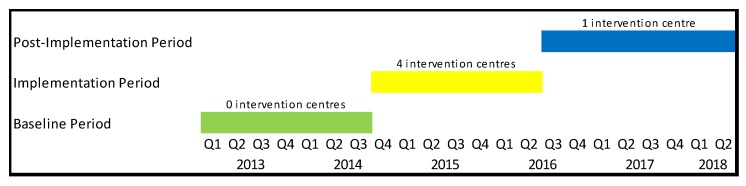
Figure enumerating the details of different intervention periods Jamshoro District, Sindh, Pakistan between Q1 2013 and Q2 2018.

We analyzed the changes in quarterly notifications of childhood TB in the district through three periods: (1) a baseline period when no resources for active TB case finding and contact tracing interventions were in place with only passive case finding with no questionnaire-based screening and contact tracing happening (January 2013 to September 2014); (2) an implementation period when active TB case finding and contact tracing interventions were deployed (October 2014 to March 2016); and (3) a post-implementation period when the project ended and additional resources for active case finding and contact tracing were withdrawn (April 2016 to June 2018) (Figure 1). We adjusted for trend in our analysis extrapolating from the baseline period. We used linear regression to calculate the effect of intervention period, a proxy for additional resources, on case notification adjusting for time.

We analyzed the changes at the specialized chest center through the baseline January 2013 to March 2015 (27 months) and implementation May 2015 to March 2016 (11 months) periods for the specialized chest center as we had phased in the implementation of the TB REACH project. Because active case finding was re-started in October 2016 at this center through Global Fund resources, the results from this center includes a post-implementation period of two quarters (Q2, 2016–Q3, 2016), and subsequent intervention quarters (Q4, 2016–Q2, 2018) that we refer to as ‘New Active Case Finding Project’.

As the specialized chest center received additional resources in the post-implementation period, a more nuanced approach is required to fully understand the trends in notification in relation to available resources. We stratified the data by center type to analyze the trends, separating the specialized chest center from the other centers in the district. We also compared the proportion of TB patients diagnosed and yield of patients diagnosed per child screened across centers during the implementation period to assess the impact by center type.

For financial analysis, we calculated the overall cost of the active case finding during the two different intervention phases at the specialized chest center. This cost did not take into account the existing government infrastructure in place. We calculated the additional cost per additional patient found by dividing our overall cost by the trend-adjusted cumulative increase in the case notifications (additional patients) at the center. All analyses were conducted using Microsoft Excel 2019 and Stata version 15 (StataCorp, College Station, TX, USA).

### 2.4. Ethical Approval

As this study used de-identified aggregated numbers from existing data sources, this study was exempted from full-review by the Institutional Review Board (IRB) of Interactive Research and Development (IRD). The TB REACH funded project was approved by the same IRB.

## 3. Results

**Figure 2:**
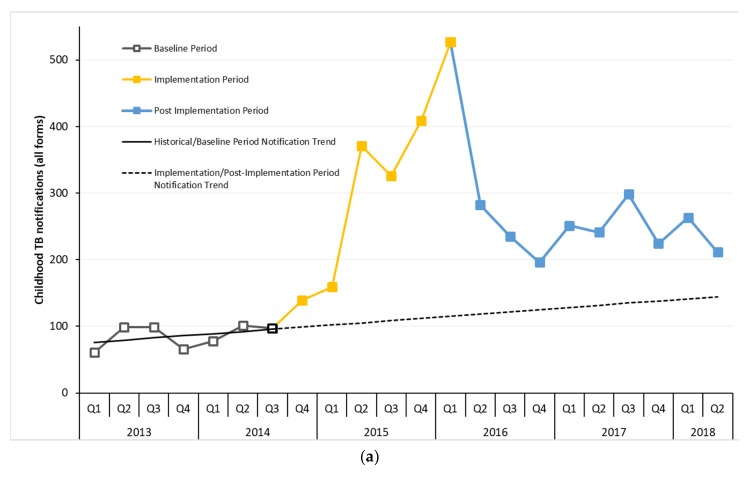

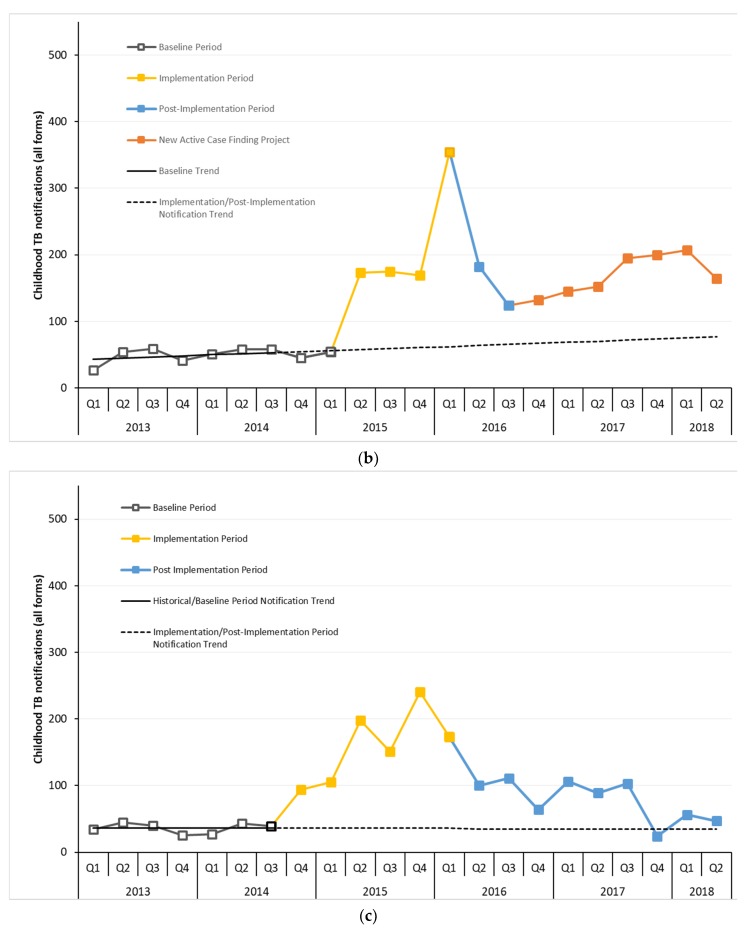
(**a**) Trend in childhood TB case notification before, during and after active case finding and contact tracing implementation in Jamshoro District, Sindh, Pakistan between Q1 2013 and Q2 2018. (**b**) Trend in childhood TB case notification before, during and after active case finding and contact tracing implementation at a specialized chest center in Jamshoro District, Sindh, Pakistan between Q1 2013 and Q2 2018. (**c**) Trend in childhood TB case notification before, during and after active case finding and contact tracing implementation at rest of the centers, in Jamshoro District, Sindh, Pakistan between Q1 2013 and Q2 2018.

The average childhood TB case notification rate in the district was 86 a quarter between quarter 1, 2013 and quarter 3, 2014 (seven quarters). It rose to an average of 322 per quarter during the six intervention quarters (quarter 4, 2014 to quarter 1, 2016), a trend-adjusted increase of 2 times (*p* < 0.01). During the nine post-implementation quarters, the average case notification was 245 per quarter, a trend-adjusted increase of 0.9 times (*p* < 0.01) (Figure 2a).

At the specialized center, the average case notification was 50 per quarter during the baseline period. In 2014, the center had screened 762 household contacts of all ages through passive contact screening with 21 contacts diagnosed with TB disease. The case notification rose steadily throughout the project implementation at the center reaching a peak of 354 children with TB in the first quarter of 2016 with an average of 218 a quarter during this period, a trend-adjusted increase of 2.6 times (*p* < 0.01). There was a fall in the case notification in quarters 2 and 3 of 2016 when no additional resources were available. Starting from quarter 4, 2016 when the new funding for active case finding started, the case notification rose again and reached 207 children with TB in quarter 1 of 2018 with an average of 172 children with TB notified a quarter during this period, a trend-adjusted increase of 1.4 times (*p* < 0.01) (Figure 2b).

The notifications in the remaining facilities in the district are depicted in Figure 2c. The average case notification was 36 a quarter during the baseline period rising to an average of 160 per quarter during the implementation period, a trend-adjusted increase of 3.9 times (*p* < 0.01). Notifications declined to an average of 78 per quarter during the post-implementation period when additional funding ceased, a trend-adjusted increase of 0.8 times (*p* = 0.07).

During the implementation period, a total of 1807 children were diagnosed with TB in the four hospitals with the specialized chest center contributing 820 (45%) of them including 188 children detected through contact tracing. The other three centers contributed 987 (55%) TB cases including 202 through contact tracing (Table 1). The yield of TB cases diagnosed per child screened from the specialized chest center was 7.5 times higher as compared to the other three centers.

Table 2 summarizes all costs incurred at the specialized chest center for the active case finding from May 2015 till March 2016 (11 months). The majority of the cost incurred (70%) was for salaries of the medical officer and community health workers hired at the center. The next biggest contributor to the cost was development of clinical decision support system (CDSS) with 18% of the total funds expended being used for it. The additional cost per additional child diagnosed was USD 41.8.

Costs incurred from October 2016 to June 2018 at the specialized chest center for active case finding in the post implementation period are also summarized in Table 2. The major cost incurred was CDSS development and maintenance cost (44%) followed by salaries of additional staff hired (35%). The additional cost per additional child diagnosed was USD 27.7.

## 4. Discussion

Studies from Pakistan, India, Nepal and Nigeria have shown that intensified case finding can result in large increases in childhood TB case notification [9,13,14,15]. Our study indicates that injection of new resources through focused active TB case finding and contact tracing efforts can substantially raise the baseline TB case notification among children. The peak case notification was reached when all aspects of the project, active case finding and contact tracing, were fully functional. Once funding for activities ceased, the district saw a marked decrease in childhood TB case notification although a residual effect of the intervention persisted with somewhat elevated notifications despite the removal of funding for new resources. Once funding for active case finding activities started again, a second increase in childhood TB case notification was observed, but the increase was smaller, likely due to limited resources available. Our findings suggest that activities that go above the routine work of the NTP require additional funding to show impact on childhood TB case diagnosis and notification.

Actively screening children for TB resulted in more than doubling of the case notification in the district during the implementation period [10] with the yield from the specialized chest center being 7.5 times higher as compared to the other three centers in this project. We believe the increased yield was due to the profile of the children presenting at the specialized center being a select population with respiratory symptoms. The cost incurred per additional child with TB found through active case finding was less than USD 42. The cost incurred per additional child with TB found through active case finding in the post implementation period was less than USD 28. The lower costs were due to the absence of active contact tracing involving greater costs from home visits.

The three non-specialized centers saw a slow decline in the case notification after the project ended, returning to baseline after almost two years. This slow decline is likely the combined result of establishment of referral behaviors, transfer-out of health center staff, programmatic and health systems strengthening and a modest communication campaign that the TB REACH project implemented. However, with time the institutional memory eroded, trained health staff left for other jobs and things returned to baseline [16,17].

Although we did not setup the evaluation as a strictly controlled trial, our results strongly point to the role that additional funding and resources play in improving performance of case detection. While not all case finding approaches will have an impact on the numbers of people being notified, a combination of different approaches including strengthening of existing systems are needed in order to improve on the status quo [9,10,18]. Most of the time, additional work will cost more money, but if the interventions are impactful, continued support must be sought [19]. As there were no notable changes in the district and the time frame is relatively short, we do not believe that external factors confound our findings.

Active TB screening in outpatient departments requires resources that NTPs do not always have. The cost of active case finding ranges from USD 72 to 963 per patient found depending on the screening algorithm used and the population being screened [20,21]. However, it will always cost more to increase the number of people offered TB services. It is estimated that in 2020 there will be a shortfall of approximately USD 6 billion globally for TB prevention, diagnosis and treatment services given the current available funding of USD 6.8 billion per year [1,22]. A significant portion of the current funding in low- and middle-income countries with high TB burdens outside of the BRICS countries is through large international donors. For example, in 19 of the 30 high burden countries more than 50% of the TB program-specific budget is through international funding [1].

Recently, the United Nations held a High-Level Meeting on Ending TB (UNHLM) where a political declaration on grounds of human rights with a target of successfully diagnosing and treating 40 million people with tuberculosis including 3.5 million children by 2022 was adopted [22]. To achieve these milestones, a greater commitment by the countries to public policies and practices related to TB will need to be realized including additional domestic funding. Increases in domestic funding has been responsible for the progress in BRICS and other European and Latin American countries in their efforts to end the TB epidemic [23]. India provides a good example where domestic funding for TB increased almost four times between 2015 and 2018 and accounts for 77% of the total TB budget of the country with no funding gap in 2019. India has seen case notifications increase by 24% nationally between 2015 and 2018 [1].

In the context of our case study, the budget for Pakistan’s national program for the year 2019 is USD 135 million with only approximately 3% of the budget funded through domestic sources and 67% of the budget remaining unfunded [1]. The proportion of domestic funding for TB Pakistan will need to increase dramatically to meet its baseline provision of services as well as provide TB screening and testing facilities at high patient-volume centers, including contact management and follow-up in all districts.

## 5. Conclusions

Children have been a historically neglected population in the TB community as they have not been sources of transmission nor can they be diagnosed with the basic tools promoted in the early years of the TB response. Global leaders, including those of Pakistan, have signed the UNHLM for TB political declaration which mandates that countries hold themselves accountable for reaching their targets. Successful, low cost, interventions such as this one that resulted in finding large numbers of missing children with TB should be scaled up with domestic funding to include health centers where patients with respiratory symptoms seek care.

## Figures and Tables

**Table 1:** (**a**) Yield of Active Case Finding in Children by Center type in Jamshoro District, Sindh, Pakistan between Q4 2014 and Q1 2016. (**b**) Yield from household contact investigation by Center type in Jamshoro District, Sindh, Pakistan between Q4 2014 and Q1 2016.

	Specialized Chest Center (%)	General Centers (%)	Total
**(a)**			
Number of children verbally screened	10,534	94,804	105,338
Children with presumptive TB	3411 (32)	2469 (3)	5880 (6)
Children tested/investigated for TB	2852 (84)	2287 (93)	5139 (87)
Children diagnosed with Bac + TB	26 (1)	16 (1)	42 (1)
Children diagnosed with All Forms TB	632 (22)	785 (34)	1417 (28)
Children with All Forms TB started on treatment	626 (99)	778 (99)	1404 (99)
**(b)**			
Number of child contacts screened	1129	1885	3014
Child contacts with presumptive TB	802 (71)	1034 (55)	1836 (61)
Child contacts/investigated for TB	707 (88)	900 (87)	1607 (88)
Child contacts diagnosed with Bac+ TB	4 (1)	1 (0.1)	5 (0.3)
Child contacts diagnosed with All Forms TB	188 (27)	202 (22)	390 (24)
Child contacts with All Forms TB started on treatment	183 (97)	202 (100)	385 (99)

**Table 2:** Cost categories at specialized chest center in Kotri through active case finding (May 2015 to June 2018).

Cost Categories	Cost for May 2015 to March 2016 in USD (%)	Cost for October 2016 to June 2018 in USD (%)
Salaries for staff at center	18,551 (70)	6649 (35)
Diagnostic tests	1344 (5)	2810 (15)
Equipment (laptop and phones)	987 (4)	798 (4)
Clinical Decision Support System development (android based application)	4658 (18)	8424 (44)
Telephone and Internet cost	572 (2)	329 (2)
Stationery and Data Management	154 (1)	100 (1)
Training of staff	142 (1)	45 (0.2)
**Total Cost**	**26,409**	**19,155**

Conversion rate for May 2015–March 2016: 1 USD = 103.1 PKR. Conversion rate for October 2016–June 2018: 1 USD = 105.3 PKR.
